# Community-level effect of the reproductive health vouchers program on out-of-pocket spending on family planning and safe motherhood services in Kenya

**DOI:** 10.1186/s12913-015-1000-3

**Published:** 2015-08-25

**Authors:** Francis Obare, Charlotte Warren, Lucy Kanya, Timothy Abuya, Ben Bellows

**Affiliations:** Reproductive Health Program, Population Council, Ralph Bunche Road, General Accident House, P.O. Box 17643, Nairobi, 00500 Kenya; Reproductive Health Program, Population Council, 4301 Connecticut Avenue, Washington, DC, NW 20008 USA; Brunel University London, Kingston Lane, Uxbridge, UB83PH London

## Abstract

**Background:**

Although vouchers can protect individuals in low-income countries from financial catastrophe and impoverishment arising from out-of-pocket expenditures on healthcare, their effectiveness in achieving this goal depends on whether both service and transport costs are subsidized as well as other factors such as service availability in a given locality and community perceptions about the quality of care. This paper examines the community-level effect of the reproductive health vouchers program on out-of-pocket expenditure on family planning, antenatal, delivery and postnatal care services in Kenya.

**Methods:**

Data are from two rounds of cross-sectional household surveys in voucher and non-voucher sites. The first survey was conducted between May 2010 and July 2011 among 2,933 women aged 15–49 years while the second survey took place between July and October 2012 among 3,094 women of similar age groups. The effect of the program on out-of-pocket expenditure is determined by difference-in-differences estimation. Analysis entails comparison of changes in proportions, means and medians as well as estimation of multivariate linear regression models with interaction terms between indicators for study site (voucher or non-voucher) and period of study (2010–2011 or 2012).

**Results:**

There were significantly greater declines in the proportions of women from voucher sites that paid for antenatal, delivery and postnatal care services at health facilities compared to those from non-voucher sites. The changes were also consistent with increased uptake of the safe motherhood voucher in intervention sites over time. There was, however, no significant difference in changes in the proportions of women from voucher and non-voucher sites that paid for family planning services. The results further show that there were significant differences in changes in the amount paid for family planning and antenatal care services by women from voucher compared to those from non-voucher sites. Although there were greater declines in the average amount paid for delivery and postnatal care services by women from voucher compared to those from non-voucher sites, the difference-in-differences estimates were not statistically significant.

**Conclusions:**

The reproductive health vouchers program in Kenya significantly contributed to reductions in the proportions of women in the community that paid out-of-pocket for safe motherhood services at health facilities.

## Background

### Introduction

In many countries, high out-of-pocket spending on healthcare services prevents some people from seeking care and can result in financial catastrophe and impoverishment for others [1–5]. The problem is particularly pronounced in low-income countries characterized by weak healthcare systems and high out-of-pocket payments due to absence of formal health insurance or other health financing schemes [6–8]. As of 2005, the World Health Organization (WHO) estimated that 44 million households worldwide faced catastrophic expenditures on healthcare (defined as expenditures comprising at least 40 % of a household’s non-subsistence income) and that 25 million households were pushed into poverty as a result [9]. Healthcare financing strategies that combine demand-side subsidies with supply-side incentives have the potential of protecting individuals in low-income countries from financial catastrophe and impoverishment arising from out-of-pocket expenditures on healthcare [5, 10–13]. The use of reproductive health vouchers is one such approach that aims to reduce the financial barriers to accessing healthcare for the poor, stimulate client demand for services, and give clients the purchasing power to seek care from the full range of available providers [10, 14–16]. Reduction in financial barriers is achieved through subsidizing the cost of services, transport to accredited providers, or both.

Effectiveness of voucher programs in reducing out-of-pocket spending for beneficiaries depends on whether both service and transport costs are subsidized as well as other factors such as service availability in a given locality and community perceptions about the quality of care. For instance, distance to care has been found to be a major determinant of uptake of health care services in developing countries [17, 18]. In such a context, voucher programs may not effectively address barriers to service utilization if they only subsidize clients’ out-of-pocket spending on health services without subsidizing transportation costs. Perceptions about the quality of available services are another key determinant of service uptake in developing countries [8, 19, 20]. It is therefore likely that even with a voucher subsidy program in place, clients might continue paying out-of-pocket at facilities that offer better services if they perceive the quality of care offered by accredited providers to be poor.

This paper examines the community-level effect of the reproductive health vouchers program on out-of-pocket spending on services in Kenya. It specifically examines the differences in changes in the likelihood of paying out-of-pocket and in the amount paid for family planning, antenatal care, delivery and postnatal care services at health facilities over time among women from voucher and non-voucher sites. Due to the voucher subsidy, we should expect a greater reduction in the likelihood of paying out-of-pocket and in the amount paid for the services by women from voucher compared to those from non-voucher sites. The premise is that as more women from voucher sites bought and used the voucher, the average out-of-pocket spending at the community level should be lower than in non-voucher sites. The increase in the number of women who did not pay for the services or who paid lower amounts in voucher sites should, in turn, be consistent with increased uptake of the voucher over time. By contrast, given the absence of the voucher subsidy in comparison sites, there should be no major change in the pool of women at the community level who did not pay or in the amount paid for the services at health facilities.

### Healthcare expenditure in Kenya

The percentage of gross domestic product (GDP) spent on healthcare in Kenya fluctuated between 4.1 % and 4.5 % over the one and half decades covering the period 1996 to 2011 (Table 1). By contrast, the percentage of GDP spent on healthcare in Uganda steadily increased over the same period from 5.5 % in 1996 to 9.5 % in 2011 while in Tanzania, the percentage of GDP spent on healthcare steadily increased between 2005 and 2011 (from 4.0 % to 7.3 %) after nearly a decade of stagnation at about 3 % (Table 1). During the same period, the Kenya government spending on healthcare as percentage of general government expenditure fluctuated between 6 % and 8 % and was constantly lower than that of her neighbours, Uganda and Tanzania (Table 1).

**Table 1:** Healthcare expenditure as percentage of gross domestic product and government spending on healthcare as percentage of general government expenditure in Kenya, Uganda and Tanzania, 1996-2011

Year	Healthcare expenditure as percentage of gross domestic product (%)			Government spending on healthcare as percentage of general government expenditure (%)		
	Kenya	Uganda	Tanzania	Kenya	Uganda	Tanzania
1996	4.1	5.5	3.4	7.3	9.8	10.2
1999	4.2	6.7	3.2	8.3	11.3	10.2
2002	4.5	7.5	3.4	8.3	9.7	11.1
2005	4.4	9.2	4.0	7.6	11.2	8.7
2008	4.2	8.8	5.4	6.1	9.5	16.0
2011	4.5	9.5	7.3	5.9	10.8	11.1

Source: World Health Organization (2013) Global Health Expenditure Database [35]

**Fig. 1:**
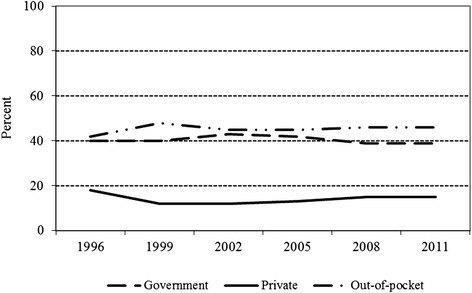
Healthcare expenditure in Kenya by source, 1996–2011. *Source*: Computed by the authors from the World Health Organization (2013) Global Health Expenditure Database

In terms of contribution to the total healthcare expenditure, out-of-pocket spending consistently comprised the largest share of total healthcare expenditure in the country over the years. In particular, the percentage of total healthcare expenditure arising from out-of-pocket payments ranged from 42 % in 1996 to 48 % in 1999 while the share of government spending ranged from 39 % in 2008 and 2011 to 43 % in 2012 (Fig. 1). Healthcare spending from other private sources, on the other hand, ranged from 12 % in 1999 and 2002 to 18 % in 1996 (Fig. 1). Available evidence shows that households in Kenya spend about 10 % of their budget on healthcare with the burden being greater among poor than rich households and for outpatient compared to inpatient services [1]. Estimates further show that 5 % of households in Kenya face catastrophic expenditures on health according to WHO definition (expenditures comprising 40 % or higher of non-subsistence income) and that about 1.5 million people in the country are pushed into poverty due to healthcare payments [1].

### Kenya reproductive health vouchers program

The reproductive health vouchers program in Kenya is implemented by the Government with major funding from the German Development Bank (KfW). The objective of the program is to reduce maternal and neonatal mortality through increased health facility delivery and improved access to appropriate health services for the poor by providing incentives for increased demand and improved service provision [21–23]. It was first piloted between 2006 and 2008 in four Counties (Kisumu, Kiambu, Kitui and Nairobi) with 54 public, private-for-profit and private-not-for-profit health facilities being accredited to provide services to voucher clients. During the second phase (2008–2011), 25 more health facilities from the same Counties were added to the program. The program was further expanded to Kilifi County in Coast region over the same period where 14 additional health facilities were accredited to provide services to voucher clients. The third phase of the program started in late 2011 and entailed accreditation of more health facilities in the same Counties.

The program subsidizes three reproductive health service components. The first component is safe motherhood services comprising four antenatal care visits, delivery care including Caesarean section if needed, postnatal care within six weeks post-delivery, and treatment of neonatal complications. The second component includes long-term family planning methods, namely, implants, intrauterine contraceptive device (IUCD), and voluntary surgical contraception. The third component comprises gender-based violence recovery services. The safe motherhood and family planning vouchers are made available through distributors in the community appointed by the voucher management agency at subsidized costs of KSh. 200 (equivalent US $2.50) and KSh. 100 (equivalent US $1.25) respectively. The beneficiaries are identified through the use of a poverty grading tool that consists of eight items on household assets and amenities, expenditure or income, and access to health services that are unique to each County. Women who score between 8 and 16 points on the tool qualify for the vouchers. The gender-based violence recovery services vouchers are, on the other hand, made freely available for clients seeking the services at accredited health facilities. Detailed descriptions of the design of the program are in Hagenmeyer et al. [21], Janisch et al. [24] and RH-OBA Technical Committee [23].

## Methods

### Study design

The study used a quasi-experimental design involving two rounds of cross-sectional household surveys in voucher and non-voucher sites. The design was chosen because there was no random assignment of sites to voucher or comparison group. Rather, voucher sites were identified by the Government in collaboration with the major funding agency based on the prevailing reproductive health indicators and availability of health facilities at the time of program inception. Health facilities in the selected sites were then approached to participate in the program and those that satisfied the accreditation criteria were contracted as voucher service providers. The comparison sites were, on the other hand, identified by the researchers in collaboration with the Ministry of Health based on geographical location (being adjacent to the intervention site), population characteristics, and availability of health facilities similar to those in voucher sites in terms of level (hospital, nursing home, health center, and dispensary) and type of ownership (public, private-for-profit and private-not-for-profit). For instance, if the intervention site had a public referral hospital, the comparison site chosen was the neighboring county that also had such a health facility. The approach was informed by the belief that neighboring counties would have populations with similar characteristics. In addition, populations living near a health facility of a certain level and type should ideally have access to the same type of health care services.

### Data

The first survey was conducted between May 2010 and July 2011 among 2,933 women aged 15–49 years while the second survey took place between July and October 2012 among 3,094 women of similar age groups. Respondents were identified from sub-locations (the smallest administrative units in Kenya) within five-kilometre radius to the health facilities that were accredited to offer services to voucher clients in four of the five program Counties (Kiambu, Kilifi, Kisumu and Kitui) and similar non-contracted facilities (in terms of level and type of ownership) in three comparison sites (Makueni, Nyandarua and Uasin Gishu Counties).

A two-stage sampling process was used. The first stage was a random sample of 14 sub-locations in each County from within five kilometres of the selected health facilities in voucher and comparison sites. Geographical positioning system (GPS) coordinates of the facilities were used to identify sub-locations that provided the sampling frame. The second stage entailed a random sample of three villages from each of the selected sub-locations. In each of the sampled villages, the local administration assisted with identifying the poorest households for inclusion in the study. Interviewers then administered the poverty grading tool that is used by the voucher management agency to target beneficiaries to the identified households to further confirm eligibility. The rationale for using the approach was used to capture as many individuals who would qualify for the vouchers as possible given that vouchers are not randomly assigned to beneficiaries. A total of 400 women (75 % poor and 25 % non-poor women for comparison) were targeted in each County in order to detect significant differences in key reproductive health indicators between voucher and comparison sites at 95 % confidence level with 80 % power [25]. More poor than non-poor women were targeted in each County in order to increase the chances of interviewing those who had actually used the voucher as opposed to simply qualifying based on the poverty grading scores.

In each selected household, women aged 15–49 years who gave birth in the past 12 months before the survey or were pregnant at the time of the interview were targeted for individual interview. In case the selected household did not have such a member, any female member of reproductive age (15–49 years) who was willing to be interviewed was approached to participate in the study. For households with two or more eligible female members, the youngest was interviewed because they are likely to be more disadvantaged in terms of accessing reproductive health services compared to older women. Respondents provided information on household assets and amenities, health-related household arrangements, food security, household expenditures on goods and services, individual background characteristics (age, education level, religious affiliation, and marital and employment status), general health status and health care utilization, childbearing experiences and intentions, as well as awareness, use and perceptions about vouchers. Women who had given birth in the five years before the survey further provided detailed information on each of the births including whether and where antenatal, delivery, and postnatal care services were sought. In the first survey, women were further asked whether they paid for safe motherhood services for the most recent birth and how much they paid. In the second survey, the questions on payments were asked for each of the births occurring in the five years preceding the survey.

Analysis of payments for safe motherhood services focuses on the most recent live birth occurring within two years before the interview in order to avoid overlap of births across surveys. A total of 951 women reported having a birth in the two years preceding the first survey (590 in voucher and 391 in non-voucher sites) while in the subsequent survey, 1,549 women reported having a birth during the reference period (915 in voucher and 634 in non-voucher sites). In both surveys, all women were asked about their knowledge and use of family planning, whether they paid for family planning services the last time they obtained a method, and how much they paid. Analysis of payments for family planning services focuses on women who used a method in the 12 months preceding the survey. The interviews were conducted in Kiswahili, English or the local language after obtaining written informed consent from respondents. The survey tool was pre-tested among a group of women with characteristics similar to those who were targeted for inclusion in the study in order to identify questions that required modification. The study obtained ethical clearance from the Institutional Review Board of the Population Council (Protocol No. 470) and the Ethics Review Committee of the Kenya Medical Research Institute (Protocol No. 174).

### Analysis

Analysis is in two parts and entails difference-in-differences estimation, that is, the difference in changes over time between women from voucher and non-voucher sites [26]. The first part is a comparison of changes in proportions of women who obtained family planning, antenatal, delivery and postnatal care services from health facilities and paid for the care they received as well as the average and median amounts paid over time (in Kenya Shillings) in voucher and non-voucher sites. The second part of the analysis involves estimation of multivariate linear regression models to examine the differences in changes in the proportions paying and the amount paid for family planning and safe motherhood services at health facilities over time between voucher and non-voucher sites. The basic model includes an interaction term between survey year and study site and adjusts for clustering of individuals at the sub-location level. The basic form of the model is specified as follows:

1 $$ {Y}_{ij}\kern0.36em ={\alpha}_0+{\alpha}_1{X_1}_{ij}+{\alpha}_2{X}_{2ij}+{\alpha}_3{X}_{1ij}*{X}_{2ij}+\dots +{X}_{ij}\beta +{\varepsilon}_j $$

The parameter *X*_*1*_ in Equation (1) is the indicator for study round, *X*_*2*_ is the indicator for study site, *X*_*ij*_ is the vector of other covariates included in the model for individual *i* from sub-location *j*, and β is the associated vector of fixed parameters. The parameter α_*0*_ represents the outcome for women from non-voucher sites at baseline (in 2010-2011); α_*1*_ is the change in the outcome between baseline and follow-up among women from non-voucher sites; α_*2*_ is the difference in the outcome between women from voucher and non-voucher sites at baseline; α_*3*_ represents the difference in the changes in the outcome between women from voucher and non-voucher sites over time (difference-in-differences estimate); and ε_*j*_ are the unobserved characteristics of women from the same sub-location that might be correlated with the outcome of interest.

Two sets of models were estimated for each of the reproductive health indicators considered, namely, family planning, antenatal, delivery and postnatal care. The first set of models had a binary outcome of whether the respondents paid for services or not while the outcome for the second set of models was the amount paid for services. The models controlled for education level, marital status, type of place of residence, duration of residence, poverty status, parity, and type of facility where services were sought. In addition, the models for safe motherhood services controlled for maternal age at the time of the most recent birth while the models for family planning services controlled for age of the respondent at the time of interview. Table 2 presents the definitions and measurement of the variables included in both models.

**Table 2:** Definition and measurement of variables used in multivariate analysis

Variable definition	Measurement
Outcome variables	
Paid for family planning services	0 = No;
	1 = Yes
Paid for antenatal care services	0 = No
	1 = Yes
Paid for delivery services	0 = No
	1 = Yes
Paid for postnatal care services	0 = No
	1 = Yes
Amount paid for family planning services	Continuous (ranges from KSh. 0 to KSh. 2,500)
Amount paid for antenatal care services	Continuous (ranges from KSh. 0 to KSh. 20,000)
Amount paid for delivery services	Continuous (ranges from KSh. 0 to KSh. 32,000)
Amount paid for postnatal care services	Continuous (ranges from KSh. 0 to KSh. 20,000)
Covariates	
Study site	0 = Non-voucher sites
	1 = Voucher sites
Study round	0 = 2010–2011 survey
	1 = 2012 survey
Study round × Study site	Interaction term between study round and study site
Maternal age at birth of child	Single years (ranges from 14 to 48); included in models for safe motherhood services
Current age of respondent	Single years (ranges from 15 to 49); included in models for family planning services
Education level	0 = No schooling/pre-unit/primary
	1 = Secondary and above
Current marital status	0 = Never/formerly married
	1 = Married/living together
Type of place of residence	0 = Urban
	1 = Rural
Duration of residence	0 = Less than 5 years/visitor
	1 = 5 years or more/always
Religious affiliation	0 = Catholic/Muslim/other
	1 = Protestant/other Christian
Poverty status^a^	0 = Non-poor (17–24 points)
	1 = Poor (8–16 points)
Parity	Ranges from 1 to 16
Place service sought	0 = Private health facility
	1 = Public health facility

^a^Based on the poverty grading tool used by the voucher management agency to identify beneficiaries; KSh: Kenya Shilling

## Results

### Characteristics of women

Across all surveys and study sites, the majority of the women interviewed were aged between 25–34 years, had primary level education, were married or living with a man at the time of the survey, were from rural areas, had lived at the place for five years or more, were poor according to the grading criteria used to identify voucher beneficiaries, and had between one and five children (Table 3). There were, however, significant variations in the distribution of women from voucher and non-voucher sites by age, highest education level, type of place of residence, and poverty status in both surveys. In particular, the proportion of women aged 15–24 years was greater in voucher than in non-voucher sites while the proportion aged 35 years and above was greater in non-voucher than in voucher sites. Similarly, the proportion of women with lower than primary level education was greater in voucher than in non-voucher sites while the proportion with secondary or higher levels of education was greater in non-voucher compared to voucher sites. The proportion living in urban areas and the proportion poor were greater in voucher than in non-voucher sites. In addition, there were significant variations in the distribution of women by duration of residence in the 2010–2011 but not in the 2012 survey.

**Table 3:** Percent distribution of women by background characteristics, survey year and study site

Characteristics	2010-2011 survey			2012 survey		
	Voucher sites (%)	Non-voucher sites (%)	*P*-value	Voucher sites (%)	Non-voucher sites (%)	*P*-value
Current age (years)			*p* < 0.01			*p* < 0.01
15-24	34.4	28.4		37.7	27.7	
25-34	44.8	45.8		43.9	47.7	
35 and above	20.4	25.9		18.0	24.6	
Don’t know	0.3	0.0		0.4	0.0	
Highest education level			*p* < 0.01			*p* < 0.01
No schooling/pre-unit	10.2	3.1		9.8	1.9	
Primary	67.3	67.9		67.0	65.4	
Secondary and above	22.5	29.0		23.2	32.7	
Current marital status			*p* = 0.16			*p* = 0.09
Never/formerly married	10.6	10.3		13.8	11.3	
Married/living together	80.3	82.5		78.6	81.5	
Formerly married	9.1	7.1		7.6	7.2	
Place of residence			*p* < 0.01			*p* < 0.01
Urban	19.4	13.0		17.5	12.7	
Rural	80.7	87.0		82.5	87.3	
Duration of residence			*p* < 0.05			*p* = 0.20
Less than 5 years/visitor	34.7	39.1		34.9	37.2	
5 years or more/always	65.3	60.9		65.1	62.8	
Religious affiliation			*p* < 0.01			*p* < 0.01
Catholic	24.7	28.6		24.9	27.1	
Protestant/other Christian	61.7	70.3		62.9	70.2	
Muslim	6.9	0.3		5.4	0.7	
Traditional/no religion	6.8	0.8		6.8	1.9	
Poverty status			*p* < 0.01			*p* < 0.01
Non-poor (17–24 points)	18.7	29.1		23.3	33.0	
Poor (8–16 points)	81.3	71.0		76.7	67.0	
Parity			*p* = 0.69			*p* = 0.42
0	4.1	3.1		5.5	4.4	
1-2	40.2	41.0		39.6	41.0	
3-4	31.9	31.6		32.4	32.5	
5 and above	23.3	23.9		22.5	22.1	
Missing	0.5	0.5		0.0	0.1	
Number of women	1,742	1,191		1,808	1,286	

Percentages may not sum to exactly 100 in some cases due to rounding; *p*-values are from Chi-square tests for differences between sites

### Use of reproductive health services

The proportions of women that had ever used any family planning method and the proportions that used a method in the 12 months preceding the survey were significantly higher in non-voucher than in voucher sites in both the 2010–2011 and 2012 surveys (Table 4). There was, however, no significant difference by study site in the proportions that had ever used the long-term methods that are subsidized by the voucher program (implants, IUCD and bilateral tubal ligation). In addition, a significantly greater proportion of women in non-voucher compared to voucher sites used the long-term methods in the 12 months preceding the 2010–2011 survey. By 2012, the proportions of women from voucher and non-voucher sites that used the methods in the 12 months preceding the survey were similar. In both surveys, most of the women in voucher and non-voucher sites who used a method in the past 12 months obtained it from a public health facility with no significant variations by study site (Table 4).

**Table 4:** Percent distribution of women by use of reproductive health services, survey year and study site

Services	2010-2011 survey		2012 survey	
	Voucher sites (%)	Non-voucher sites (%)	Voucher sites (%)	Non-voucher sites (%)
Ever used family planning	(*N* = 1,742)	(*N* = 1,191)	(*N* = 1,808)	(*N* = 1,286)
Used any method	59.6	75.2^**^	66.9	75.0^**^
Used long-term method^a^	7.6	9.6	13.8	13.6
Used FP last 12 months	(*N* = 1,742)	(*N* = 1,191)	(*N* = 1,808)	(*N* = 1,286)
Used any method	39.2	54.5^**^	47.9	52.6^**^
Used long-term method^a^	5.3	7.1^*^	10.1	10.3
Source of last FP method^b^	(*N* = 683)	(*N* = 649)	(*N* = 866)	(*N* = 676)
Public health facility	79.7	76.9	13.4	16.0
Private health facility	14.4	15.6	71.4	70.7
Other/missing	6.0	7.6	15.2	13.3
Ever use of voucher	(*N* = 1,742)	(*N* = 1,191)	(*N* = 1,808)	(*N* = 1,286)
Used safe motherhood	15.4	0.0^**^	43.9	0.0^**^
Used family planning	1.8	0.0^**^	6.6	0.0^**^
Used gender-based violence	0.0	0.0	0.0	0.0
Used any voucher	16.0	0.0^**^	45.0	0.0^**^
Sought antenatal care for most recent birth	100.0(*N* = 588)	100.0(*N* = 361)	96.6(*N* = 912)	96.5(*N* = 632)
Source of antenatal care	(*N* = 588)	(*N* = 361)	(*N* = 881)	(*N* = 610)
Public health facility	85.0	87.5	78.9	89.0^**^
Private health facility	14.0	11.9	20.7	11.0^**^
Home/other/missing	1.0	0.6	0.5	0.0
Source of delivery care	(*N* = 588)	(*N* = 361)	(*N* = 912)	(*N* = 632)
Public health facility	39.0	41.0	42.3	43.5
Private health facility	14.1	12.7	21.9	13.0^**^
Home/other/missing	46.9	46.2	35.8	43.5^**^
Source of postnatal care	(*N* = 588)	(*N* = 361)	(*N* = 912)	(*N* = 632)
Public health facility	42.4	43.2	60.2	68.2^**^
Private health facility	13.8	11.6	22.5	13.9^**^
Home/other/missing	43.9	45.2	17.3	17.9

^a^Include methods that are subsidized by the voucher program (implants, intrauterine contraceptive device and female sterilization); ^b^Among those who used a method in the last 12 months; FP: Family planning; *p < 0.05; **p < 0.01

As expected, none of the women in non-voucher sites had ever used any of the reproductive health vouchers in either 2010–2011 or 2012. By contrast, the proportion of women in voucher sites that had used the safe motherhood voucher increased from 15 % in the 2010–2011 survey to 44 % in the 2012 survey. Similarly, the proportion that had ever used the family planning voucher increased from 2 % in 2010–2011 to 7 % in 2012. In both surveys, none of the women from voucher sites reported having ever used the gender-based violence recovery services voucher. It could be that women felt stigmatized if they reported using the gender-based violence recovery services voucher which may have contributed to underreporting or lack of awareness about the voucher given that it was only available at the facility level.

Results in Table 4 further show that nearly all women who had a birth in the two years preceding the survey sought antenatal care with no significant variations by study site. There were no significant variations in the source of antenatal, delivery and postnatal care services for women from voucher and non-voucher sites in 2010–2011. In 2012, however, the proportions of women that obtained antenatal, delivery and postnatal care services from private health facilities were significantly higher among those from voucher compared to those from non-voucher sites. The proportions of women that obtained antenatal and postnatal care services from public health facilities were, on the other hand, significantly higher in non-voucher than in voucher sites. For delivery care, there was no significant difference in the proportions of women from voucher and non-voucher sites that obtained the services from public health facilities (Table 4).

### Changes in payment patterns for services

Overall, there was a greater reduction in the proportions that paid for family planning, antenatal, delivery and postnatal care services in voucher than in non-voucher sites (Table 5). In absolute terms, the differences in changes between voucher and non-voucher sites were greater for safe motherhood than for family planning services. With respect to amounts paid, there was no change in the average and median amount that women paid for family planning services in voucher sites over time. In non-voucher sites, the average amount paid for family planning services increased by KSh. 32 (representing a 52 % increase) while the median amount remained unchanged at KSh. 50. The average amount paid for antenatal care services declined by KSh. 27 (20 % decline) in voucher sites while the median amount declined from KSh. 50 in 2010–2011 to zero in 2012 (100 % decline). By contrast, the average amount paid for antenatal care in non-voucher sites increased by KSh. 139 while the median amount increased by KSh. 50 over time (representing 78 % and 40 % increase respectively).

**Table 5:** Changes in payment patterns for reproductive health services at facilities by study site and survey year

Indicator	Voucher sites			Non-voucher sites		
	2010-2011 survey	2012 survey	Change	2010-2011 survey	2012 survey	Change
Proportions paying (%)						
Family planning	73.8(*N* = 598)	71.9(*N* = 750)	−1.9	86.3(*N* = 546)	85.2(*N* = 568)	−1.1
Antenatal care	69.1(*N* = 433)	42.0(*N* = 877)	−27.1	85.0(*N* = 267)	85.6(*N* = 610)	0.6
Delivery care	66.1(*N* = 221)	30.6(*N* = 586)	−35.5	92.1(*N* = 140)	89.6(*N* = 357)	−2.5
Postnatal care	27.8(*N* = 209)	7.3(*N* = 754)	−20.5	26.1(*N* = 138)	18.1(*N* = 519)	−8.0
Mean amount paid (KSh)						
Family planning	50.00(*N* = 598)	50.00(*N* = 750)	0.00	61.00(*N* = 546)	93.00(*N* = 568)	32.00
Antenatal care	136.00(*N* = 429)	109.00(*N* = 876)	−27.00	178(*N* = 266)	317.00(*N* = 606)	139.00
Delivery care	2,047.00(*N* = 215)	980.00(*N* = 581)	−1,067.00	3,193.00(*N* = 133)	2,696.00(*N* = 352)	−497.00
Postnatal care	174.00(*N* = 204)	18.00(*N* = 754)	−156.00	151.00(*N* = 137)	78.00(*N* = 518)	−73.00
Median amount paid (KSh)						
Family planning	30.00(*N* = 598)	30.00(*N* = 750)	0	50.00(*N* = 546)	50.00(*N* = 568)	0.00
Antenatal care	50.00(*N* = 429)	0.00(*N* = 876)	−50.00	125.00(*N* = 266)	175.00(*N* = 606)	50.00
Delivery care	500.00(*N* = 215)	0.00(*N* = 581)	−500.00	2,000.00(*N* = 133)	1,500.00(*N* = 352)	−500.00
Postnatal care	0.00(*N* = 204)	0.00(*N* = 754)	0.00	0.00(*N* = 137)	0.00(*N* = 518)	0.00

Non-zero positive values indicate an increase in the estimates over time; KSh: Kenya Shilling (1 USD ≈ KSh. 86)

The decline in the average amount paid for delivery and postnatal care services was more than twice greater in voucher than in non-voucher sites (Table 5). In particular, the average amount paid for delivery services declined by KSh. 1,067 and KSh. 497 in voucher and non-voucher sites respectively (representing 52 % and 16 % decline respectively). Similarly, the average amount paid for postnatal care services declined by KSh. 156 and KSh. 73 in voucher and non-voucher sites respectively (90 % and 40 % decline respectively). The median amount paid for delivery services declined from KSh. 500 to zero (100 % decline) in voucher sites and by 25 % in non-voucher sites (from KSh. 2,000 to KSh. 1,500). By contrast, the median amount paid for postnatal care services remained unchanged at zero in both voucher and non-voucher sites.

### Difference-in-differences estimates

The results from the multivariate linear regression models with the difference-in-differences estimates for the proportions paying for various reproductive health services at facilities in voucher and non-voucher sites over time are presented in Table 6. The difference-in-differences estimates were statistically significant for the proportions paying for antenatal (*p* < 0.01), delivery (*p* < 0.01) and postnatal care (*p* < 0.05) but not for family planning services (*p* = 0.75). The results further show that the proportions that paid for family planning services significantly declined with higher parity (*p* < 0.05). In addition, the proportions that paid for antenatal and delivery care services were significantly greater among those who sought services from public than from private health facilities (*p* < 0.05 and *p* < 0.01 respectively). There was, however, no significant difference in the proportions paying for the various reproductive health services by poverty status (Table 6).

**Table 6:** Coefficient estimates from multivariate regression models for proportions of women paying for reproductive health services at health facilities

Covariates	Family planning	Antenatal care	Delivery care	Postnatal care
Study site (voucher sites = 1)	−0.12^**^(−0.18; −0.06)	0.16^**^(−0.25; −0.06)	−0.27^**^(−0.37; −0.17)	−0.00(−0.11; 0.11)
Study round (2012 survey = 1)	−0.01(−0.06; 0.04)	0.01(−0.05; 0.06)	−0.04(−0.10; 0.02)	−0.09^*^(−0.17; −0.01)
Study round × Study site	−0.01(−0.08; 0.06)	−0.27^**^(−0.36; −0.18)	−0.30^**^(−0.40; 0.19)	−0.12^*^(−0.22; −0.02)
Current age (single years)	0.00(−0.00; 0.01)	n/a	n/a	n/a
Maternal age at last birth (single years)	n/a	0.00(−0.01; 0.01)	0.00(−0.00; 0.01)	−0.00(−0.01; 0.00)
Highest education level (secondary and above = 1)	−0.02(−0.06; 0.02)	0.01(−0.04; 0.06)	0.12(−0.03; 0.01)	−0.04(−0.09; 0.00)
Current marital status (married/ living together = 1)	0.05^*^(0.00; 0.11)	0.04(−0.01; 0.09)	0.02(−0.04; 0.08)	−0.04(−0.09; 0.02)
Type of place of residence (rural = 1)	0.02(−0.07; 0.12)	−0.07(−0.18; 0.03)	−0.06(−0.19; 0.07)	−0.06(−0.13; 0.02)
Duration of residence (5 or more years/always = 1)	−0.02(−0.06; 0.02)	−0.02(−0.06; 0.02)	−0.02(−0.07; 0.03)	0.01(−0.03; 0.05)
Religious affiliation (Protestant/ other Christian = 1)	0.02(−0.02; 0.06)	0.01(−0.03; 0.05)	0.01(−0.04; 0.06)	0.01(−0.03; 0.05)
Poverty status (poor = 1)	−0.01(−0.05; 0.03)	−0.00(−0.04; 0.04)	0.01(−0.04; 0.06)	0.02(−0.06; 0.02)
Parity	−0.02^**^(−0.04; −0.01)	0.00(−0.01; 0.02)	−0.02(−0.04; 0.00)	0.01(−0.00; 0.02)
Facility type (public = 1)	−0.05^*^(−0.10; −0.00)	0.10^*^(0.02; 0.18)	0.21^**^(0.15; 0.28)	−0.01(−0.06; 0.04)
Constant	0.90^**^(0.76; 1.05)	0.78^**^(0.61; 0.95)	0.75^**^(0.54; 0.95)	0.41^**^(0.23; 0.58)
Number of cases	2,461	2,179	1,302	1,618

Estimates are based on Equation (1) in the text; n/a: not applicable; 95 % confidence intervals are in parentheses; *p < 0.05; **p < 0.01

Table 7 presents the results from the multivariate linear regression models with the difference-in-differences estimates for the amount paid for reproductive health services at facilities in voucher and non-voucher sites over time. The estimates for the differences in changes in the amount paid for services were statistically significant for family planning and antenatal care (*p* < 0.01 in each case) but not for delivery (*p* = 0.11) and postnatal care services (*p* = 0.45). Other results show that the amount paid for antenatal and delivery care services significantly declined with parity (*p* < 0.01 in each case). In addition, the amount paid for family planning, antenatal and delivery care services was significantly lower among women who sought services from public than from private health facilities (Table 7). Women with at least secondary level education significantly paid more for family planning and delivery care than those with lower levels of education while poor women significantly paid less for delivery and postnatal care services compared to their non-poor counterparts.

**Table 7:** Coefficient estimates from multivariate regression models for amount paid for reproductive health services at health facilities

Covariates	Family planning	Antenatal care	Delivery care	Postnatal care
Study site (voucher sites = 1)	−7.23(−19.82; 5.23)	−41.16(−85.39; 3.06)	−844.79(−1701.30; 11.71)	70.39(−207.79; 348.57)
Study round (2012 survey = 1)	32.63^**^(13.90; 51.37)	140.47^**^(65.13; 215.80)	−335.19(−1119.16; 448.79)	−41.61(−224.02; 140.80)
Study round × Study site	−33.43^**^(−54.97; 11.88)	−172.05^**^(−252.65; −91.45)	−726.53(−1624.88; 171.83)	−103.93(−376.60; 168.74)
Current age (single years)	1.10(−0.27; 2.28)	n/a	n/a	n/a
Maternal age at last birth (single years)	n/a	2.79(−0.21; 6.15)	111.02^**^(52.26; 169.79)	9.30(−13.05; 31.66)
Highest education level (secondary and above = 1)	22.11^**^(5.74; 38.48)	7.28(−42.27; 56.84)	600.90^*^(126.53; 1075.27)	58.32(17.99; 134.64)
Current marital status (married/ living together = 1)	3.27(−8.06; 14.59)	47.82(−5.83; 101.47)	169.07(−354.16; 692.31)	−69.51(−212.27; 73.25)
Type of place of residence (rural = 1)	8.21(−0.84; 17.26)	−55.54(−124.28; 13.19)	−303.86(−978.63; 370.92)	37.37(−48.88; 123.62)
Duration of residence (5 or more years/always = 1)	0.47(−9.78; 10.72)	60.40(−10.27; 131.08)	−160.04(−597.01; 276.93)	7.60(−51.26; 66.46)
Religious affiliation (Protestant/ other Christian = 1)	−0.96(−14.89; 12.97)	5.48(−39.61; 50.56)	13.60(−343.87; 371.08)	50.15^*^(4.74; 95.56)
Poverty status (poor = 1)	2.12(−10.10; 14.34)	−31.70(−79.88; 16.49)	−455.64^*^(−888.01; −23.27)	−112.22^*^(−212.86; −11.57)
Parity	−0.17(−5.50; 5.17)	−24.52^**^(−40.12; −8.93)	−325.94^**^(−485.99; −165.89)	−26.59(−82.13; 28.94)
Facility type (public = 1)	−43.74^**^(−63.88; −23.59)	−90.07^**^(−148.14; −32.00)	−638.31^*^(−1238.26; −38.35)	−11.23(−101.89; 79.43)
Constant	50.44^**^(22.17; 78.70)	244.07^**^(131.94; 356.20)	1715.90^*^(61.13; 3370.68)	3.19(−379.66; 386.05)
Number of cases	2,461	2,169	1,279	1,611

Estimates are based on Equation (1) in the text; n/a: not applicable; 95 % confidence intervals are in parentheses; *p < 0.05; **p < 0.01

## Discussion

The major finding of this paper is that over time, there were significantly greater declines in the proportions of women from voucher sites that paid for antenatal, delivery and postnatal care services at health facilities compared to those from non-voucher sites. The changes were also consistent with increased uptake of the safe motherhood voucher over time in voucher sites, which nearly tripled between 2010–2011 and 2012. There was, however, no significant difference in changes in the proportions of women from voucher and non-voucher sites that paid for family planning services. Although the proportion of women in voucher sites that had ever used the family planning voucher more than tripled between the two surveys, the uptake of the voucher was substantially lower compared to the safe motherhood voucher. The findings therefore indicate that the reproductive health vouchers program significantly reduced the proportions of women in the community that paid out of pocket for safe motherhood services. The findings further suggest that significant reductions in the proportions of women in the community paying for reproductive health services can be achieved through strategies aimed at increasing the uptake of the vouchers. Such strategies include intensive marketing campaigns, proper targeting of clients, controlling potential fraud, widening the range of services that are subsidized, increasing the number of accredited facilities to ensure ease of access to services, and effectively monitoring and improving quality of care through practice [10, 27].

The second major finding of the paper is that there were significant differences in changes in the amount paid for family planning and antenatal care services by women from voucher sites compared to those from non-voucher sites. The average amount paid for family planning services by women from voucher sites remained unchanged while the average amount paid for antenatal care declined over time. By contrast, the average amount paid for the two services by women from non-voucher sites increased over time. The significance of the difference-in-differences estimates for these outcomes could therefore be due to the variations in trends in the average amount paid for the services. Although there were greater declines in the average amount paid for delivery and postnatal care services by women from voucher sites compared to those from non-voucher sites, the difference-in-differences estimates were not statistically significant. Ideally, voucher clients should not pay anything for services that are subsidized by the program. This was corroborated by the data which showed that the median amount paid for the services by women who had ever used the vouchers was zero and that none of the women who had ever used the voucher paid for services when they used it (not shown). It could therefore be that in both surveys, women from voucher sites who paid for delivery and postnatal care services were largely non-beneficiaries of the program.

The findings of the paper are consistent with the view in the literature that demand-side subsidies combined with supply-side incentives have the potential to protect economically disadvantaged individuals from financial catastrophe and impoverishment arising from out-of-pocket expenditures on health care services [5, 10–13]. However, substantial reductions in out-of-pocket expenditure for voucher beneficiaries can be achieved if the programs subsidize both transport and service costs as is the case with the Bangladesh maternal health voucher scheme [28, 29]. Findings from the Kenya voucher program, for instance, show that some women who purchased the vouchers failed to use them because transportation costs to accredited health facilities were higher than service costs at nearby non-contracted providers [27, 30].

The other strategies that governments in developing countries have adopted to cushion poor households from catastrophic out-of-pocket expenditure on health services are abolition of user fees and conditional cash transfers. For instance, the Kenya Government abolished user fees for safe motherhood services at public health facilities from June 2013 [31]. However, available evidence suggests that although removal of user fees increases service uptake, it may have a negative impact on the quality of care [32]. In addition, the effectiveness of conditional cash transfer programs in settings such as that of sub-Saharan Africa may be affected by supply side barriers [33]. Unlike abolition of user fees and conditional cash transfers, vouchers aim to improve service delivery through explicit performance-based contracting with service providers based on set minimum standards of care as well as through stimulating competition for voucher clients [10, 15]. In addition, vouchers not only empower clients to seek services but also generate revenue for health facilities which can be used to improve service quality.

The above findings might, however, be influenced by the study’s limitations. First, there was no random assignment of facilities, villages or clients to the voucher program. It could therefore be argued that any differences between voucher and non-voucher sites could be due to unobserved differences in respondent characteristics. Second, the identification of respondents from within specific geographical distances to contracted facilities might lead to under- or over-representation of voucher users depending on how spread they are from the facilities. This could, in turn, result in under- or over-estimation of the community-level effect of the program on out-of-pocket expenditure on reproductive health services at facilities. Under-estimation may result from under-representation of voucher clients while over-estimation may arise if voucher clients were over-represented in the sampled areas. Third, the effect of the program could be undermined by periods of low voucher sales especially between November 2008 and May 2009 which were due to delays in finalization of contracts and printing of vouchers as the program transitioned from pilot to scale-up phase [34]. The low sales might have affected the number of beneficiaries, hence limiting the impact of the program at the community level.

## Conclusion

Despite the limitations, the findings of this paper suggest that the reproductive health vouchers program in Kenya significantly contributed to reductions in the proportions of women in the community that paid out-of-pocket for safe motherhood services in the regions where it was implemented.

## Abbreviations

GDP: Gross domestic product
GPS: Global positioning system
IUCD: Intra-uterine contraceptive device
KEMRI: Kenya Medical Research Institute
KfW: Kreditanstaldt fuer Wiederaufbau (German Development Bank)
KSh: Kenya Shilling
NCST: National Council for Science and Technology
NCPD: National Council for Population and Development
OBA: Output-based aid
RH: Reproductive health
WHO: World Health Organization
